# Compensatory regrowth of the mouse bladder after partial cystectomy

**DOI:** 10.1371/journal.pone.0206436

**Published:** 2018-11-26

**Authors:** Grace B. Delos Santos, Megan Y. Devine, Jessica Wetterlin, Paula R. Firmiss, Natalie A. Kukulka, Diana K. Bowen, Edward M. Gong, Robert W. Dettman

**Affiliations:** 1 Loyola University Health System, Department of Urology, Maywood, Illinois, United States of America; 2 Developmental Biology, Stanley Manne Children’s Research Institute, Anne and Robert H. Lurie Children’s Hospital of Chicago, Chicago, Illinois, United States of America; 3 Northwestern University, Feinberg School of Medicine, Department of Urology, Chicago, Illinois, United States of America; Centro Nacional de Investigaciones Oncologicas, SPAIN

## Abstract

Cystectomy is the removal of all or part of the urinary bladder. It has been observed that there is significant regrowth of the bladder after partial cystectomy and this has been proposed to be through regeneration of the organ. Regrowth of tissue in mammals has been proposed to involve compensatory mechanisms that share many characteristics of true regeneration, like the growth of specialized structures such as blood vessels or nerves. However, the overall structure of the normal organ is not achieved. Here we tested if bladder growth after subtotal cystectomy (STC, removal of 50% of the bladder) was compensatory or regenerative. To do this we subjected adult female mouse bladders to STC and assessed regrowth using several established cellular parameters including histological, gene expression, cytokine accumulation and cell proliferation studies. Bladder function was analyzed using cystometry and the voiding stain on paper (VSOP) technique. We found that STC bladders were able to increase their ability to hold urine with the majority of volume restoration occurring within the first two weeks. Regenerating bladders had thinner walls with less mean muscle thickness, and they showed increased collagen deposition at the incision as well as throughout the bladder wall suggesting that fibrosis was occurring. Cell populations differed in their response to injury with urothelial regeneration complete by day 7, but stromal and detrusor muscle still incomplete after 8wks. Cells incorporated EdU when administered at the time of surgery and tracing of EdU positive cells over time indicated that many newborn cells originate at the incision and move mediolaterally. Basal urothelial cells and bladder mesenchymal stem cells but not smooth muscle cells significantly incorporated EdU after STC. Since anti-inflammatory cytokines play a role in regeneration, we analyzed expressed cytokines and found that no anti-inflammatory cytokines were present in the bladder 1wk after STC. Our findings suggest that bladder regrowth after cystectomy is compensatory and functions to increase the volume that the bladder can hold. This finding sets the stage for understanding how the bladder responds to cystectomy and how this can be improved in patients after suffering bladder injury.

## Introduction

Tissue regeneration is a process by which an injured or amputated anatomic structure regrows to form a normally functioning appendage or organ. This process has been observed in animals for hundreds of years and is understood to occur in some but not all organisms [1]. While some organisms such as echinoderms, crustaceans, amphibians and fish have the ability to completely regenerate tissues such as limbs, fins and heart, mammals demonstrate limited regeneration typically accompanied by scar formation [2, 3]. This form of regeneration is termed compensatory growth and is necessitated by physiologic requirements of the entire body, as is seen, for example, in the compensatory growth of one kidney when the other is removed [4].

In mice and humans, limb regeneration is limited to the distal digit and this capacity is greater in juvenile animals [5, 6]. The same has been observed in the heart, where regeneration after removal of the apex only occurs in neonatal mouse pups [7]. Even so, some scarring has been reported in injured neonatal hearts [8]. Of the internal organs that redevelop after removal or injury, the liver and urinary bladder have demonstrated the greatest ability to regrow. The liver has a relatively low rate of addition of new cells during normal life, but after up to 75% resection (hepatectomy), the remaining organ can rapidly regrow to restore the full weight of the liver [9, 10]. Since individual lobes of the liver do not regrow after hepatectomy, liver regeneration is considered to be compensatory growth, rather than a process that restores the original form of the organ [4]. Still, multiple cell-types regrow in the liver after hepatectomy including blood vessels and nerves, so at the cellular level, regeneration is occurring [11].

Another example of mammalian regeneration is the urinary bladder. It has been reported for decades that the bladder regrows even if 75% of the tissue is removed [12, 13]. Like the heart and digit tip, the ability of the bladder to regrow after cystectomy is affected by age [14]. Interestingly, in animal models, resected bladders that have been augmented with segments of extracellular matrix demonstrate rapid recellularization and restored bladder function [15]. Thus, cells resident to the bladder contribute to the recellularization process supporting the idea that the bladder has a regenerative capacity.

We wondered if bladder regeneration represented a true regenerative process or regeneration accompanied by scarring. Our hypothesis tested was that bladder regeneration after cystectomy involves scar formation and that this limits bladder function after cystectomy. We tested this in mice subjected to subtotal cystectomy (STC). Here we found that bladders grew after STC, were capable of storing increasing volumes of urine but this never became equivalent to control mice that had undergone laparotomy. Urothelium rapidly regenerated to line the bladder, but the surrounding sub-mucosal and detrusor muscle layers took longer to grow. Growth originated from the incision and cells moved towards the bladder neck. STC bladders had increased collagen deposition and were characterized by both regeneration associated and fibrosis associated gene expression. Immune cells infiltrated the incision site and STC bladders expressed pro-inflammatory but not anti-inflammatory cytokines. Together, these results favored our hypothesis that bladder regeneration is accompanied by fibrosis and scarring, which limits bladder function after regrowth.

## Materials and methods

### Experimental animals

The Institutional Animal Care and Use Committee of the Stanley Manne Children’s Research Institute approved all animal studies. Adult CD1 female mice (Jackson Labs) were used in all studies.

### Subtotal cystectomy procedure

Subtotal cystectomy (STC) was performed on adult female CD1 mice. Anesthesia was induced using 3% vaporized isoflurane (Baxter) at a flow rate of 2.5 L/min. Mice were then transferred to the operating table, placed in a supine position, and kept under anesthesia using a rodent anesthesia mask to deliver a continuous flow of isoflurane. The lower abdomen was shaved and prepared with povidone-iodine solution. A low-midline incision was made and carried down through the abdominal wall. The bladder was identified. The superior 50% of the bladder was sharply excised. The bladder was then closed with 6–0 monofilament polypropylene suture in a running fashion. For “linear incision” surgery a single incision through the bladder wall into the lumen was made with scissors, urine expressed and closed as above. The muscle and skin layers were then closed with 6–0 vicryl suture in a running fashion. Mice that underwent only low-midline laparotomy served as “sham” controls. Post-operatively, mice recovered in a cage that was placed on a heating pad until normal ambulation was observed. All mice received 500 mg acetaminophen crushed and mixed into drinking water. All mice were observed twice daily for the first seven days and once daily afterwards. If any signs of distress were observed including hunched posturing, decreased ambulation, and/or ruffled fur, the mice were immediately euthanized.

### Voiding stain on paper (VSOP)

VSOPs were obtained from mice at various time points postoperatively. Mice were separated into individual metabolic cages with underlying Whatman filter paper, given unrestricted access to food and water, and observed for 2 hours. The filter paper was then imaged using UV light. Number and pattern of voids was noted, and maximum voided volume calculated.

### Quantitative PCR

Quantitative PCR was performed according to methods previously described [16]. RNA was isolated from whole bladders removed at the bladder neck and ureters dissected. Ct values were recorded on the Applied Biosystems qRTPCR machine and converted into ΔCt values. Statistical significance was determined by comparing ΔCt values for the tested gene to ΔCt values for the internal control gene: GAPDH. Data is presented in two forms: tables with ΔCt values, standard error of the mean and statistical significance as determined using unpaired Student’s T-Tests; and bar graphs with asterisks indicating statistical significance (P < 0.05) as indicated in S2–S5 Tables. Error bars for the ΔΔCt bar graphs were calculated using error propagation [17].

### Cytokine array

STC and sham operated animals were prepared as above (8 STC and 8 sham). Bladders were harvested one week postoperatively. Sample collection was performed according to product instructions.

Homogenates in PBS with protease inhibitors were prepared from 4 bladders, creating two pooled samples for STC and two pooled samples for sham operated. After homogenization, Triton X-100 was added to a final concentration of 1%. Samples were frozen at -80C, thawed, and centrifuged at 10,000*x*g for 5 minutes to remove cellular debris. Quantification of sample protein concentrations using a total protein assay was performed. Proteome Profiler Array Mouse Cytokine Array Panel A (Catalog Number ARY006, R&D Systems) was used. Pooled samples were added to array membranes, washed and prepared as directed. Four total membranes were used, two for STC and two for sham. Membranes were placed on an autoradiography film and exposed to X-ray film for 1, 5, and 10 minutes. Spot intensity was quantified using FIJI (ImageJ v2.0.0). In this process, the autoradiograph was scanned by transmission light scanning on a flatbed scanner. The image was imported into FIJI and converted to an 8-bit image. First, background was subtracted (process, subtract background) and then inverted (edit, invert). Spots were measured using an ROI of constant area and integrated density values were measured from each spot. Each blot contains two spots for each cytokine, thus allowing us to carryout duplicates. Values were exported to Excel for analysis. Fold increase was calculated by dividing the mean integrated density values for STC data points by the integrated density values for sham operated data points.

### Histology

Paraffin embedding, sectioning and staining were performed by the Mouse Histology and Phenotyping Core at Northwestern University. Slides were stained with Hematoxylin and Eosin or Masson’s Trichrome stain according to standard procedures.

### Immunofluorescence of mouse bladders

Whole fixed bladders were utilized. Sections of 10–12 μm were cut for all experiments. Section staining was performed as previously described [16]. A complete list of primary and secondary antibodies can be found in S1 Table.

### Tracing cell movement with EdU

To trace the movement of cells that divided during days 3–7 post-STC we used a method adapted from Mercer et al., 2013 [18]. Here, STC or sham surgeries were performed as above and 100 μL 1mM 5-ethynyl-2’-deoxyuridine (EdU, Invitrogen) in 70% phosphate buffered saline (PBS, Cellgro) was injected once intraperitoneally. Four days later EdU incorporation was “chased” by injecting 100μL 100mM thymidine (Sigma) in 70% PBS. Bladders were harvested at designated time points, embedded in OCT, frozen and stored at -80°C. EdU incorporation was detected in 10μm sections using the Click-IT EdU Assay according to the manufacturer’s instructions (Invitrogen). EdU was quantified using Adobe Photoshop v12.

### Renal function

At sacrifice blood was taken by cardiac puncture and blood was sent for blood urea nitrogen (BUN) and creatinine levels (Antech Diagnostics, Irvine CA, USA).

### Statistical analyses

All statistical analyses were done using GraphPad Prism version 5 for Mac OSX, (GraphPad Software, La Jolla, CA) or Excel (Microsoft).

## Results

### Bladder function is not fully restored after STC

**Fig 1:**
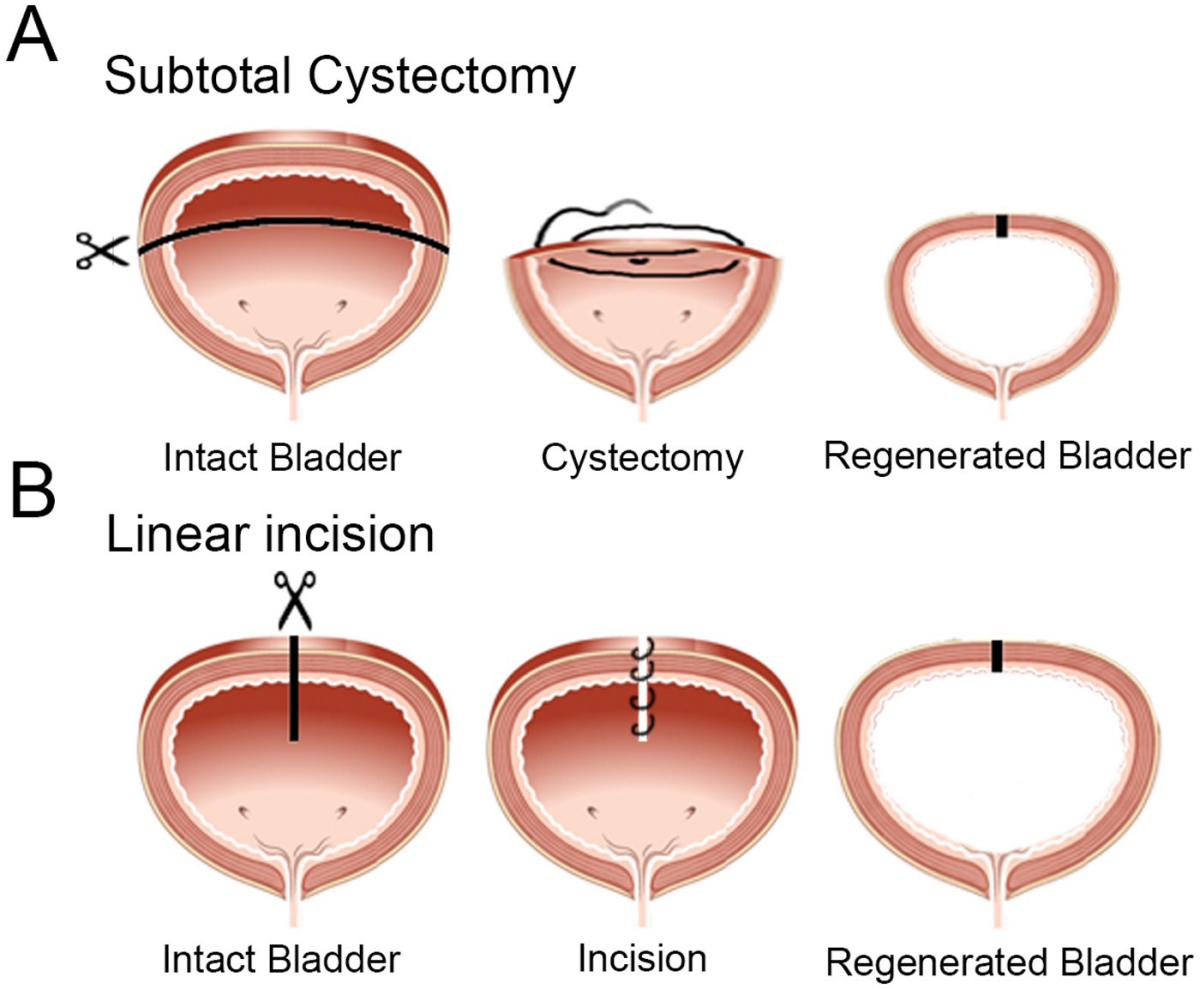
Strategy of the STC and subsequent analysis. (A) Schematic outlining how the STC surgery was performed and sutured. (B) Schematic indicating how the linear incision surgery was performed and sutured.

We carried out a partial cystectomy (~50%) in female mice as a model of bladder regeneration. Female mice were chosen as previous studies of bladder regeneration after partial cystectomy in rodents were carried out in females [13, 14, 19, 20]. Two control surgeries were used. The first control incision was a “linear incision” surgery in which a linear incision was made through the bladder into the lumen, after which the incision was sutured without and removal of bladder tissue. The second control was a “sham” surgery in which a laparotomy was performed. Here the bladder was manipulated in a similar manner to that in the subtotal cystectomy surgery, before it was placed back in the abdomen and the abdominal wall sutured. Our approaches are shown in Fig 1.

**Fig 2:**
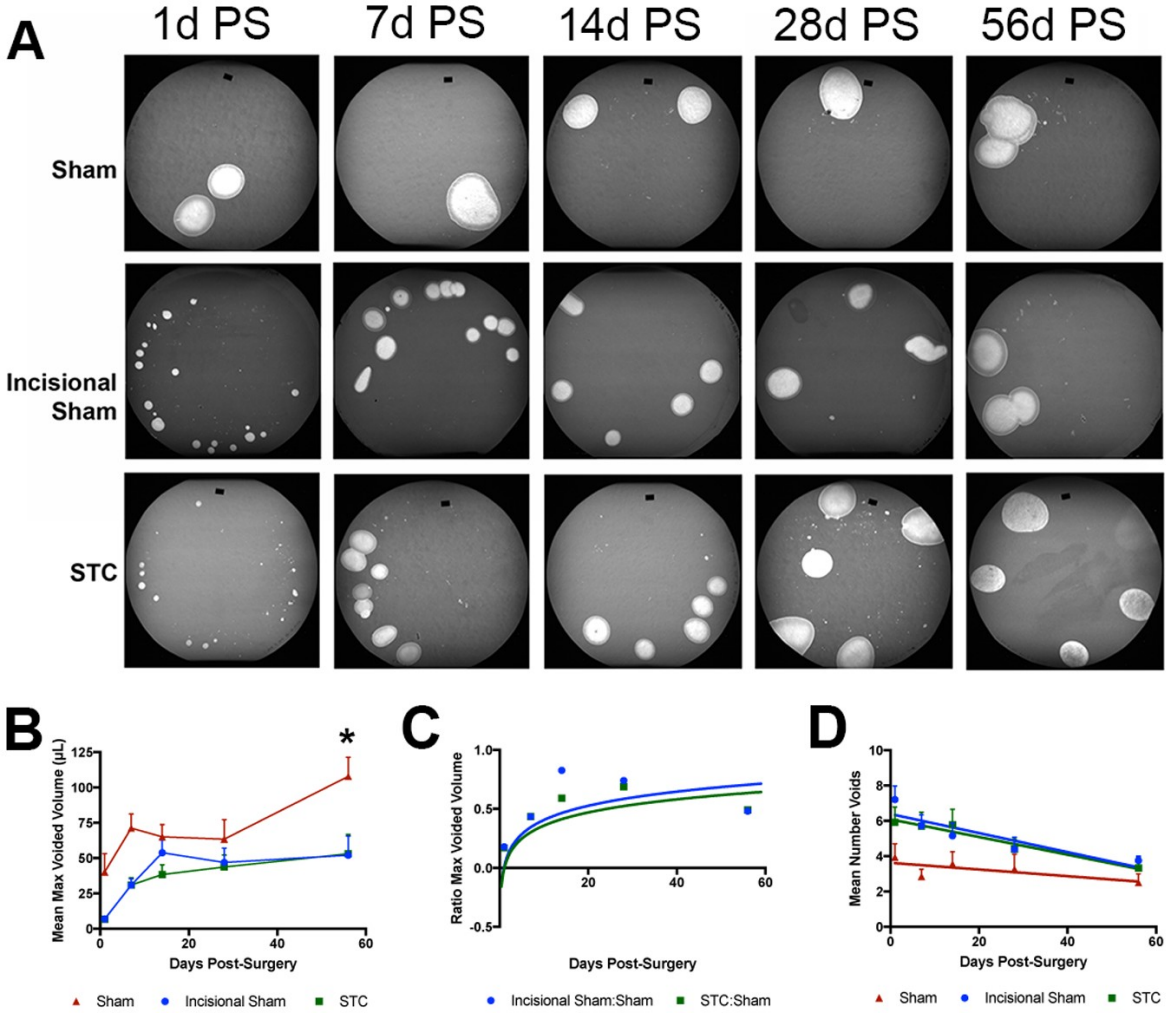
Recovery of voiding volume after STC. (A) Representative images of VSOP performed on sham, incisional sham and STC bladders, 1d PS, 1wk (7d PS), 2wk (14d PS), 4wk (28d PS) and 8wk (56d PS) after surgery. (B) Mean maximum voiding volume determined from VSOP analysis after determining a standard curve using mouse urine. Graph demonstrating results for maximum voided volume per void in linear incision (blue line) STC (green line) and sham (red line) bladders. The y-axis indicates mean volume in μL and the x-axis indicates the time after surgery that mice were subjected to VSOP in days. Error bars are standard error of the mean (SEM). (C) Graph of ratio of maximum voided values in (B) for linear incision to sham (blue line) and STC to sham surgery (green line). By 56d PS the volume of STC bladders had reached 63% of age-matched sham control bladders. (D) Graph indicating the mean number of voids as indicated by spots on the blotting paper after 2h. Error bars are SEM.

Mice were allowed to recover from each surgery and were analyzed using a voiding stain on paper (VSOP) assay in metabolic cages [21]. Sham surgery mice demonstrated voiding patterns similar to non-surgically altered mice and sham mice in our other studies with partial bladder outlet obstruction [16, 22]. Here we found that mice subjected to linear incision and STC demonstrated abnormal voiding stain patterns as compared with sham surgery mice (Fig 2A). Upon quantifying VSOP assays we found that mean voided volumes were lower in both linear incision and STC mice relative to shams (Fig 2B). Mean voided volume was initially much lower in linear incision and STC mice. Voiding volumes increased in linear incision and STC mice with the maximum volumes observed at 56d PS (8wk). While sham animals exhibited somewhat variable mean voided volumes, they were always higher than either linear incision or STC mice and were significantly different than shams at 8wk (Fig 2B). We calculated the ratio of the mean voided volumes and found that linear incision and STC bladders gradually recovered their ability to hold urine. This reached a maximum of 63% of sham at 8wk for both linear incision and STC (Fig 2C). We recorded the number of voids that were observed on the blotting paper in a 2h period (Fig 2D). In the first week after surgery linear incision and STC mice had a higher voiding frequency than sham mice (Fig 2A and 2D). As recovery continued, voiding frequency declined so that at 8wk, shams, on average, voided twice in 2h and STCs voided three times (Fig 2D). To further analyze the recovery of the bladder after STC we carried out cystometry under anesthesia for mice that had recovered for various times after surgery. Here, we observed that the STC bladders had higher filling, higher maximum voiding and resting pressures than sham operated controls (S1 Fig). Together, VSOP and cystometry indicated that in STC mice, the ability of the bladder to function as a reservoir is restored after STC, but not to levels equivalent to sham operated bladders. Post-mortem blood urea nitrogen (BUN) and creatinine levels did not significantly vary between the experimental groups indicating that kidney function was not compromised. Since, the linear incision surgery appeared to alter the bladder as much as STC, we continued our study using shams as controls in most cases.

### Regrowth of the bladder after STC involves increased collagen deposition

**Fig 3:**
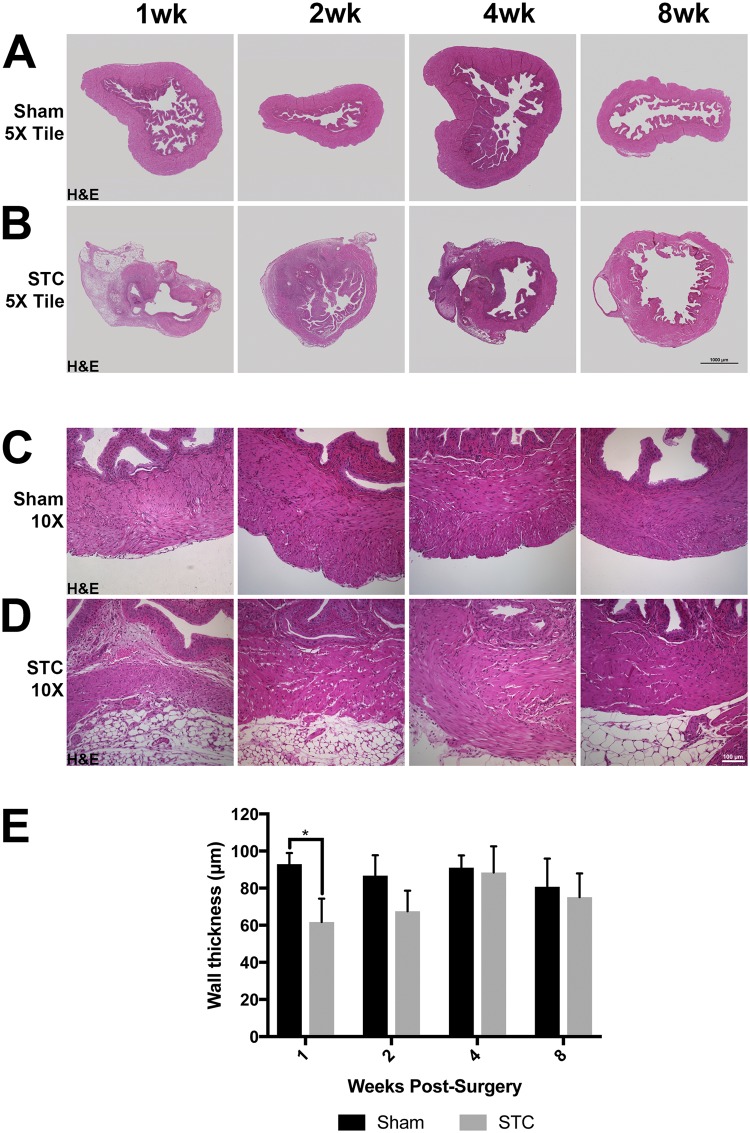
Bladder wall thickness and collagen deposition are altered after STC. (A-D) Representative images of sham operated (A, C) and STC-operated (B, D) bladders 1wk (7d PS), 2wk (14d PS), 4wk (28d PS) and 8wk (56d PS) after surgery stained with hematoxylin and eosin (H&E). (E) Bar graph indicating mean wall thickness in μm. Black bars represent sham operated bladders and gray bars represent STC operated bars. Error bars represent SEM. Statistical significance (P < 0.05) is indicated by an asterisk.

**Fig 4:**
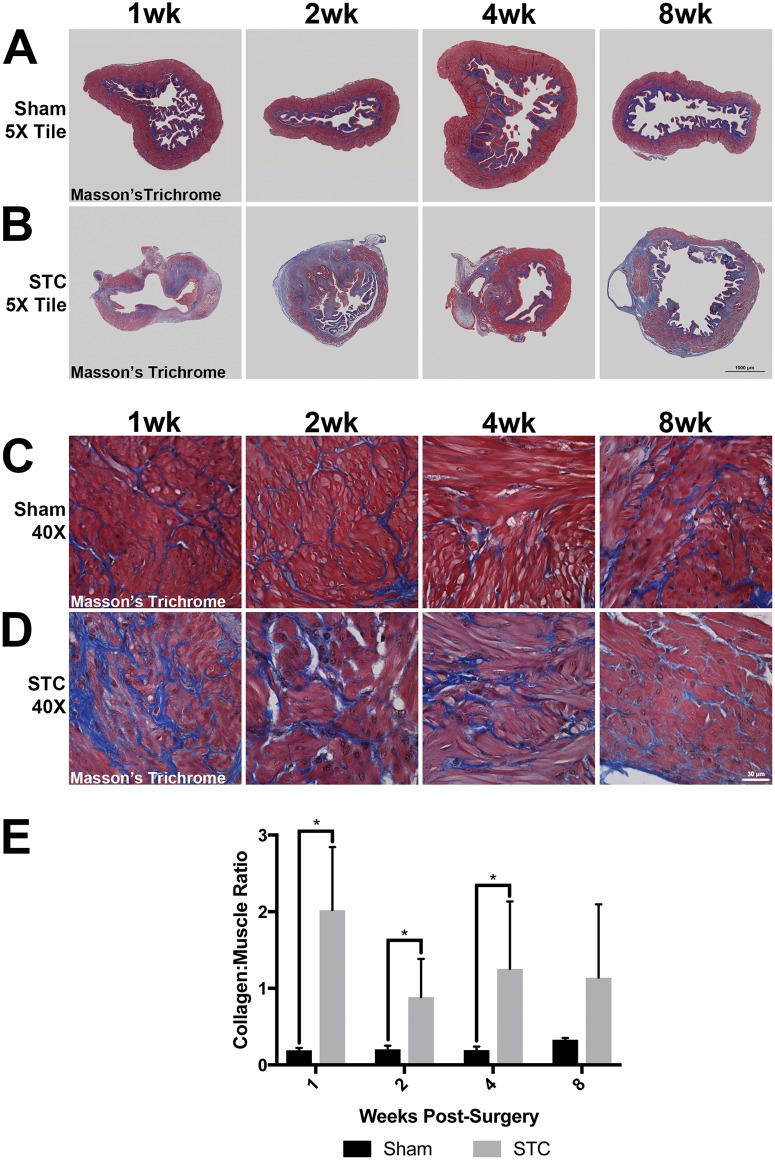
Increased collagen to smooth muscle ratio in STC bladders. (A-D) Representative images of sham operated (A, C) and STC-operated (B, D) bladders 1wk (7d PS), 2wk (14d PS), 4wk (28d PS) and 8wk (56d PS) after surgery stained with Masson’s trichrome stain. (E) Bar graph indicating the collagen to smooth muscle ratio in sham and STC bladders at the same time points. Black bars represent sham operated bladders and gray bars represent STC operated bars. Error bars represent SEM. Statistical significance (P < 0.05) is indicated by asterisks.

To investigate if the bladder wall was fully restored after STC, we examined sections of STC and sham bladders up to 8wk after cystectomy using histology. Sections were either stained with hematoxylin and eosin (Fig 3H & 3E) or Masson’s Trichrome for collagen (Fig 4). To determine if the detrusor muscle was the same thickness as in the shams we measured wall thickness at multiple locations and determined the mean wall thickness in sham and STC bladders (Fig 3E). Here we observed that at 7d PS (1wk) regenerating bladders had significantly thinner walls with less mean muscle thickness than shams. At 14d PS (2wk) the mean wall thickness was still thinner than sham operated controls, but this difference was not statistically significant.

Upon Masson’s Trichrome staining, we observed that collagen deposition was increased in STC bladders as compared to shams. Whereas wall thickness was restored by 4wks, the muscle layer did not regrow completely in areas distal to the suture site (Fig 4B). Instead, this area was populated cells with a mesenchymal appearance surrounded by collagen accumulation. In sham bladders, the sub-mucosal stroma contained organized collagen fibers and the detrusor muscle had discrete areas that reacted with trichome stain (Fig 4A and 4C). STC bladders showed increased collagen deposition near the suture site as well as throughout the bladder wall (Fig 4B and 4D). This included large areas near the suture site and bladder dome that reacted with trichome stain (Fig 4B). We quantified collagen accumulation as determined by trichrome stain at various times after surgery and found that in sham operated bladders, collagen represented approximately 20% of the area of the section. In STC bladders, collagen staining represented from 40–60% of the area of the section. When we analyzed the area distal to the suture site, collagen staining represented 60–85% of the tissue (Fig 4C). Thus, collagen accumulated in STC bladders to a greater degree than in sham bladders, with its highest accumulation occurring in the bladder where the dome once existed.

**Fig 5:**
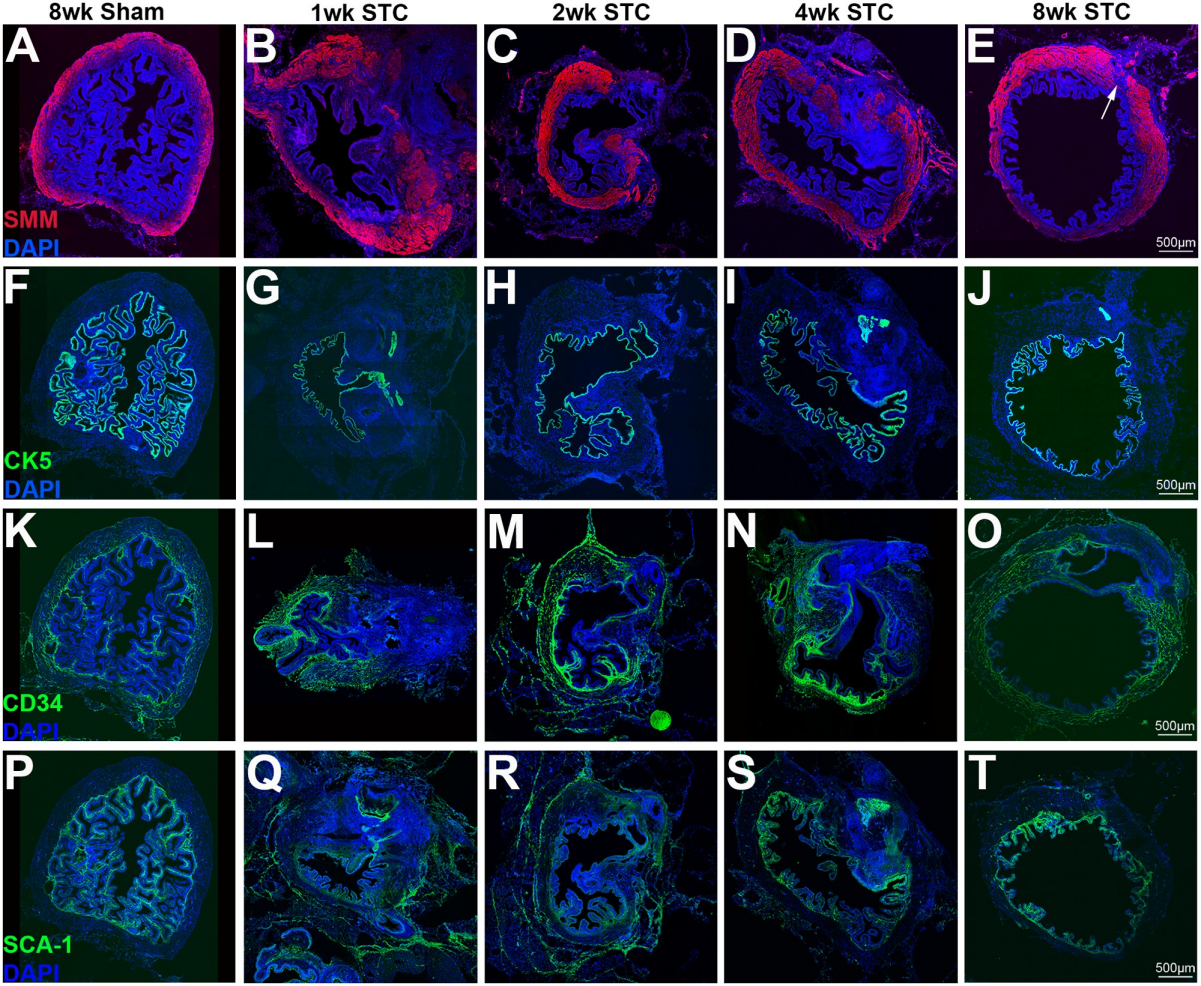
Regrowth of the detrusor muscle 8wk after STC surgery. Tiled confocal images of sham operated and STC operated bladders 1wk (7d PS), 2wk (14d PS), 4wk (28d PS) and 8wk (56d PS) after surgery. (A-E) The top row of panels shows staining for smooth muscle myosin (SMM) in red with nuclei counterstained in blue (DAPI). (F-J) Row of panels shows staining for cytokeratin-5 (CK5) marking the urothelium (green). (K-O) Row of panels shows staining for CD34 marking BMSCs (green). (P-T) Row of panels shows staining for Sca-1 marking BMSCs (green). Magnification bars are 500μm and are shown in the lower portion of panels (E, J, O, T). Arrow in (E) points to gap in smooth muscle regrowth at 8wk.

To examine the regrowth of specific cell-types in the bladder we stained sections with antibodies to cytokeratin-5 (CK5, urothelium), CD34 and Sca-1 (submucosal stroma) and smooth muscle myosin (SMM, smooth muscle). Cell populations differed in their response to injury. Smooth muscle was present from the bladder neck up through the suture site, but even at 8wk PS there was typically a gap in smooth muscle staining at the bladder dome (Fig 5B–5E). Urothelial regrowth was rapid and by 7d PS was contiguously lining the lumen of the bladder, even underneath the suture site (Fig 5G–5J). CD34 and Sca-1 was disorganized in the 1-4wk samples, appearing both near the bladder neck as well as in isolated areas of the re-growing dome (Fig 5L–5O and 5Q–5T). By 8wk, both CD34 and Sca-1 has reorganized into the submucosal stroma (Fig 5O and 5T). We stained bladder sections 1wk PS with an antibody to keratin-14 (Krt14), which marks a population of basal urothelial progenitor cells that begin to differentiate into all urothelial cell types after injury [23]. We found that cells expressed Krt14 in sham operated bladders and that the number of cells expressing Krt14 was similar to that in linear incision bladders. Krt14 staining of basal urothelial progenitor cells in STC bladders appeared higher than either sham or linear incision (S2 Fig). This was consistent with a rapid repopulation of the entire bladder with urothelium after STC.

**Fig 6:**
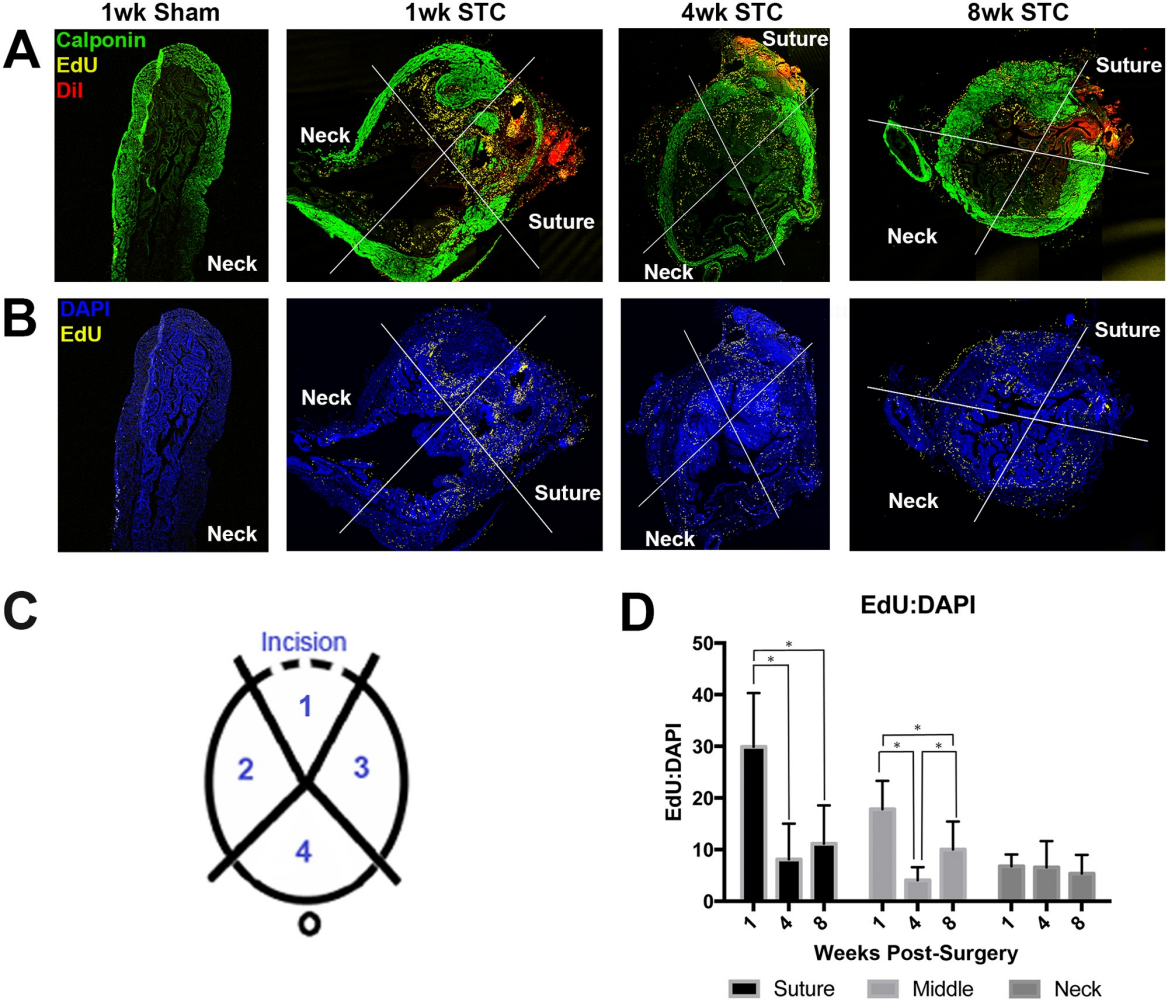
Regenerating cells originate primarily from the suture site. (A) Tiled confocal images of bladder sections stained with anti-calponin (green), EdU (yellow), and DiI (red). Time after STC surgery is indicated above each set of panels. The white lines forming a cross indicate the regions that were analyzed for fluorescent pixels. DiI was applied to the suture immediately after the bladder was sealed. (B) The same images in (A) showing only EdU (yellow) and DAPI (blue) pixels. This view allows us to see the EdU pixels clearly. (C) EdU incorporation in a sham operated bladder at 1wk showing little incorporation of EdU. (D) Quantification of pixels in images such as those shown in (A). The bar graph indicates the ratio of EdU pixels to DAPI pixels in the indicated regions at the indicated times after surgery. Significance is indicated above bars (P < 0.05). Error bars are standard error of the mean.

To examine if blood vessels extend into the regenerated bladder dome we stained whole bladders with an antibody to PECAM (CD31). In non-operated and sham bladders blood vessels appeared as an organized network (Fig 6A, 6B, 6E and 6F). In linear incision and STC bladders analyzed at 28d PS (4wk), blood vessels were found throughout the regenerated part of the bladder (center) indicating that angiogenesis occurs during the regenerative process. However, these blood vessels appeared less organized than in the non-operated or sham bladders. This confirmed that blood vessel growth occurs in the region of the regenerate consistent with a regenerative model.

### Regrowth of the bladder originates from the suture site

**Fig 7:**
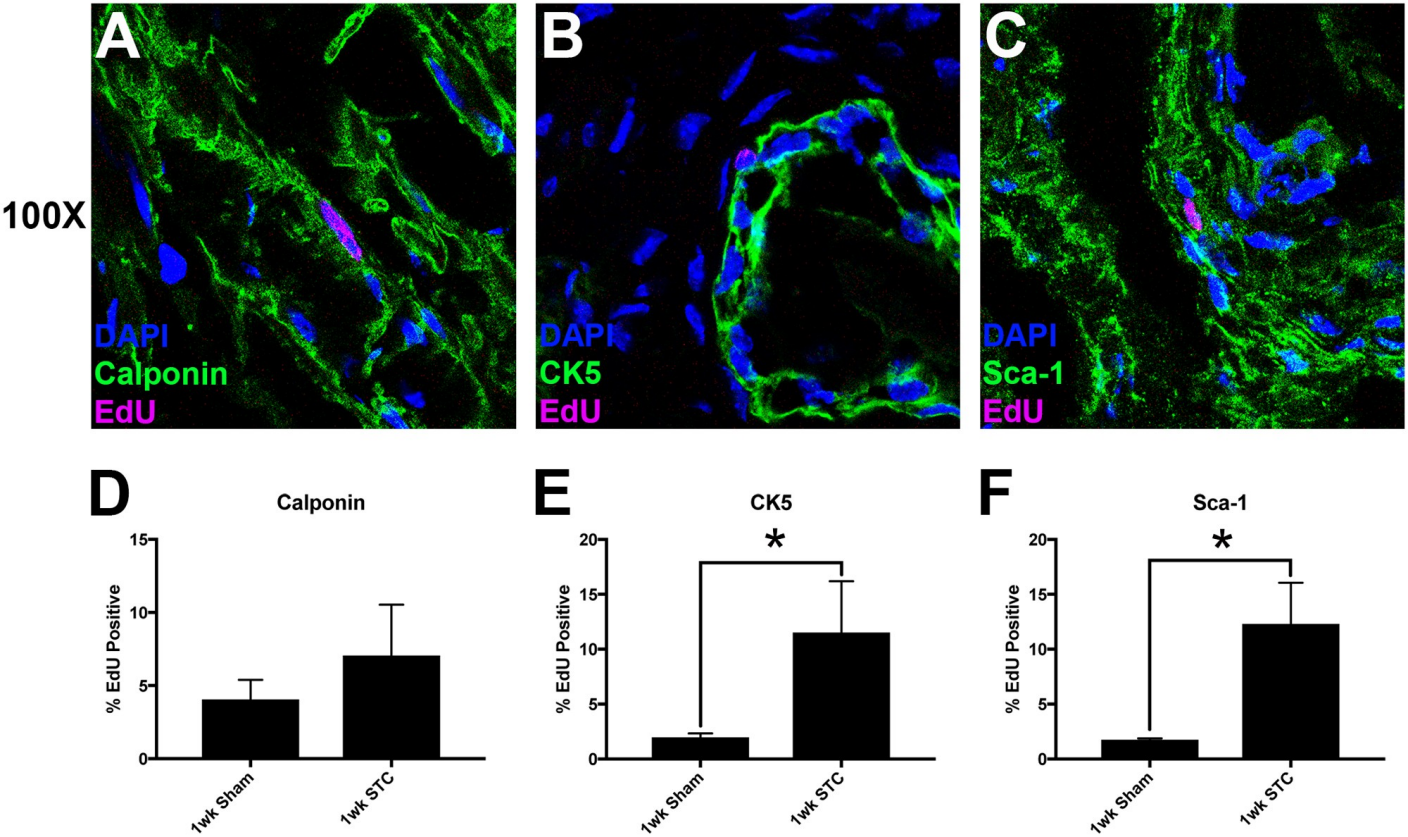
Quantification of EdU incorporation in smooth muscle, urothelial and BMSCs. (A-C) Representative confocal images of EdU treated STC bladders stained with anti-calponin (A), anti-CK5 (B) and anti-Sca-1 (C) all in green. All nuclei are stained with DAPI (blue) and EdU incorporation is shown in pink. Quantification of co-localization of EdU^+^ nuclei with antibody stain. This is indicated on the y-axis as %EdU incorporated. The number shown is the mean number of %EdU^+^/antibody^+^ cells divided by the total number of antibody^+^ cells x100 per section counted. Significance (*) is indicated above bars (P < 0.05). Error bars are standard error of the mean.

We investigated cell proliferation after STC by staining sections with antibodies to phospho-histone H3 (PHH3). In adult sham bladders, we observed increased staining with anti-PHH3, suggesting that, at baseline, a small number of cells are actively dividing at the time the bladders are fixed (S3 Fig). This was surprising as increased cell division was reported to be a response to STC in rats [13]. However, since PHH3 is detectable only from late G2 to late anaphase, many cells that may have divided in response to STC may be missed. We therefore stained sections with anti-Ki-67 and this confirmed that many cells were, in fact, dividing after STC (S4 Fig). To follow the movement of dividing cells after STC we pulse labeled them with the enduring marker EdU 3d after STC. This nucleotide analog is incorporated during the cell cycle and persists in cells for weeks [18]. Thymidine was injected 4d after the EdU injection at a 100-fold higher concentration to “chase” the EdU from injected mice. Bladders were either harvested at the time of the chase (7d) or at 2, 4 and 8wk PS. At 1wk, few EdU^+^ cells were observed in sham bladder sections (Fig 7C). In 1wk STC bladders we observed large numbers of cells that had absorbed EdU indicating that many cells divided 3–7 days after STC (Fig 7A and 7B).

We scored the position of EdU pixels within four zones of the regenerating bladder: the area near the suture site (zone 1, suture); the areas proximal and lateral to the suture site (zones 2 and 3, mediolateral), and the area nearest the bladder neck (zone 4, neck) (Fig 7C). In this experiment, the suture site was simultaneously labeled with DiI, a red fluorescent lipophilic dye (Fig 7A). We observed that after 1wk, ~30% percent of the nuclear DAPI pixels (blue) overlapped EdU pixels (yellow) in zone 1. This was significantly different after 4wks, when ~8% of the nuclear DAPI pixels overlapped EdU pixels (Fig 7D). At 8wk, this number rose to ~12%, but while this was significantly different than 1wk, it was not significantly different than 4wk. Thus, there was a net loss of the cells labeled by EdU in zone 1 after STC. In the mediolateral zones 2 and 3, ~18% of the nuclear DAPI pixels overlapped EdU pixels by 1wk. This also changed significantly by 4wk, with EdU pixels dropping to ~5% of DAPI pixels. Curiously, the percent of nuclear EdU pixels significantly increased in mediolateral zones at 8wk suggesting that there is net movement of labeled cells into these regions of the bladder from 4-8wks. The fewest number of EdU pixels were observed in the bladder neck region (zone 4). In 1wk bladders ~8% of the nuclear DAPI pixels overlapped EdU pixels. This number essentially remained constant at 4wk and 8wk. Some of the changes in EdU pixels can be attributed to net loss of EdU labeling during the 7wk time course, as ~56% of the nuclear pixels overlapped EdU pixels in 1wk STC bladders but this number dropped to only ~28% by 8wk (Fig 7D). This indicated that the EdU label was metabolized by the mice over time. In summary, EdU pulse labeling indicated that large numbers of cells were dividing after STC surgery and while the EdU was slowly metabolized, there was net movement of EdU labeling from the suture site to the mediolateral parts of the bladder.

**Fig 8:**
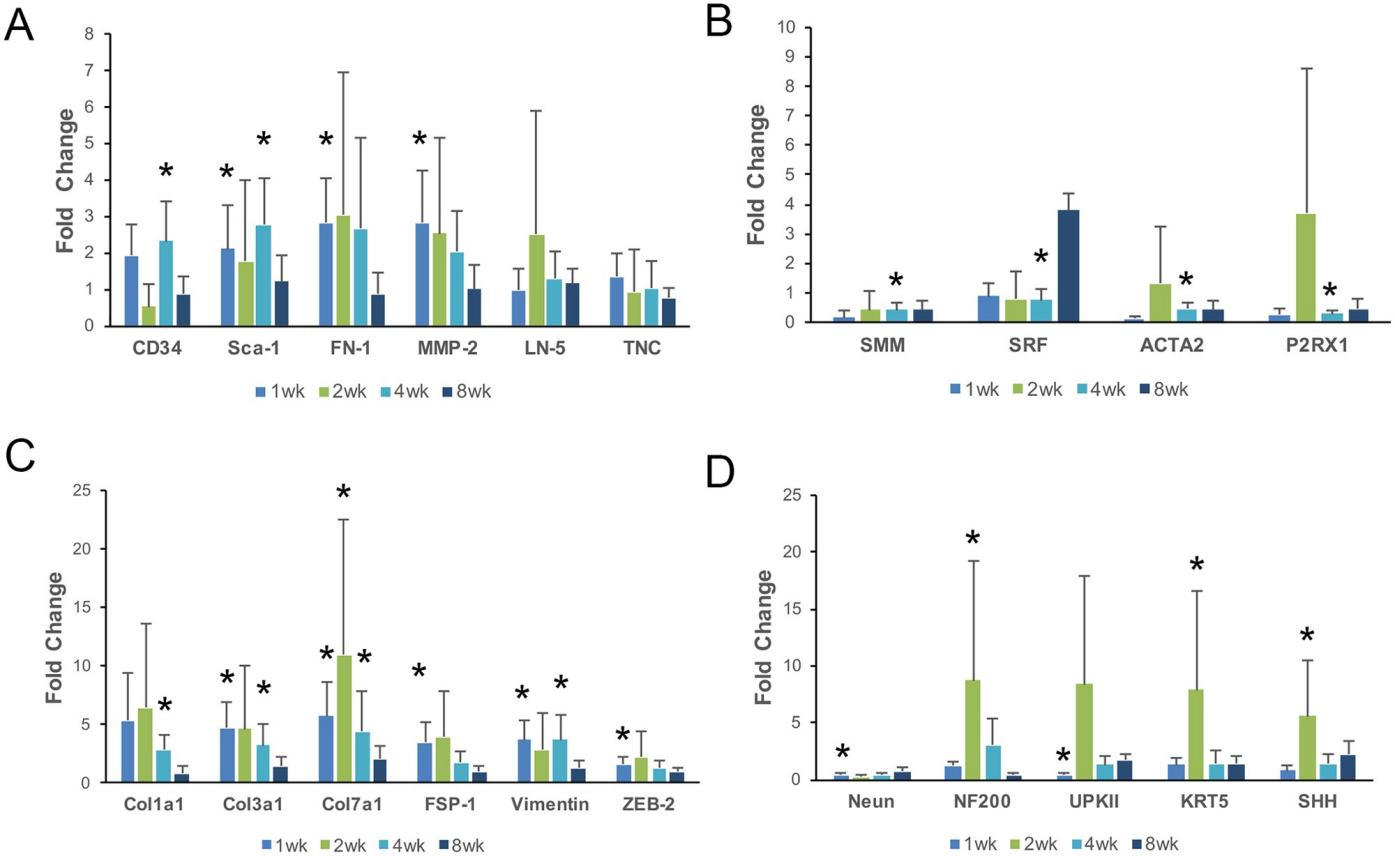
Transcripts associated with regeneration and scarring are expressed in the STC bladder. (A-D) Quantitative polymerase chain reaction (QPCR) analysis of total RNA isolated from sham operated or STC operated bladders 1wk (7d PS, light blue bars), 2wk (14d PS, green bars), 4 wk, (28d PS, aqua bars) and 8wk (56d PS, dark blue bars). All bars were standardized to the level of GAPDH transcripts in each sample. Bars represent the fold-change relative to sham for each gene. (A) Quantification of representative stem cell and tissue remodeling transcripts: Abbreviations are as follows: CD34-sialomucin; Sca-1-stem cell antigen 1; FN1-fibronectin; MMP2-matrix metalloproteinase 2; LN5-laminin 5; and TNC-tenascin C. (B) Quantification of representative detrusor muscle transcripts: SMM-smooth muscle myosin; SRF-serum response factor; ACTA2-smooth muscle alpha actinin; and P2RX1-purinergic receptor P2X1. (C) Quantification of representative fibrosis transcripts: Col1α1-collagen1 alpha1; Col3α1- collagen3 alpha1; Col7α1-collagen7 alpha1, FSP-1-fibroblast specific protein-1; Vim-vimentin; and ZEB2-zinc finger E-box binding homeobox-2. (D) Quantification of representative neural and urothelial transcripts: NeuN- Hexaribonucleotide Binding Protein-3, NF200-neurofilament 200; UpkII-uroplakin II; Ck5- cytokeratin-5; and SHH-sonic hedgehog. Fold change on graph indicates log2 fold change as determined by the ΔΔCt method. Data for the graphs is found in S2–S5 Tables. Statistical significance was determined by comparing the distribution of ΔCt values for shams versus STC surgery using unpaired Student’s T-Tests. Error bars on ΔΔCt values were calculated using error propagation. Asterisks indicate statistical significance (P < 0.05) as indicated in S2–S5 Tables.

To investigate which cells were dividing primarily in response to STC we co-stained 1wk STC bladders labeled with EdU 3d PS with antibodies to calponin (smooth muscle cells), CK5 (urothelial cells) and Sca-1 (bladder mesenchymal stem cells, aka BMSCs). We counted the proportion of cells that were labeled by each antibody that contained an EdU^+^ nucleus in both sham and STC bladders (Fig 8). Here we found that all three cell types incorporated EdU after STC. Smooth muscle incorporated a modest amount of EdU, but this was not statistically significant (Fig 8D). Basal urothelial cells (Fig 8B and 8E) and BMSCs (Fig 8C and 8F) increased cell proliferation significantly after STC. Increased proliferation of urothelial cells was consistent with the observation that urothelium was rapidly reformed within the regenerate (Fig 5G and 5H). Increased cell division in BSMCs indicates that these cells may be involved in the regenerative response to STC.

### Genes associated with regeneration and scarring are expressed after STC

**Fig 9:**
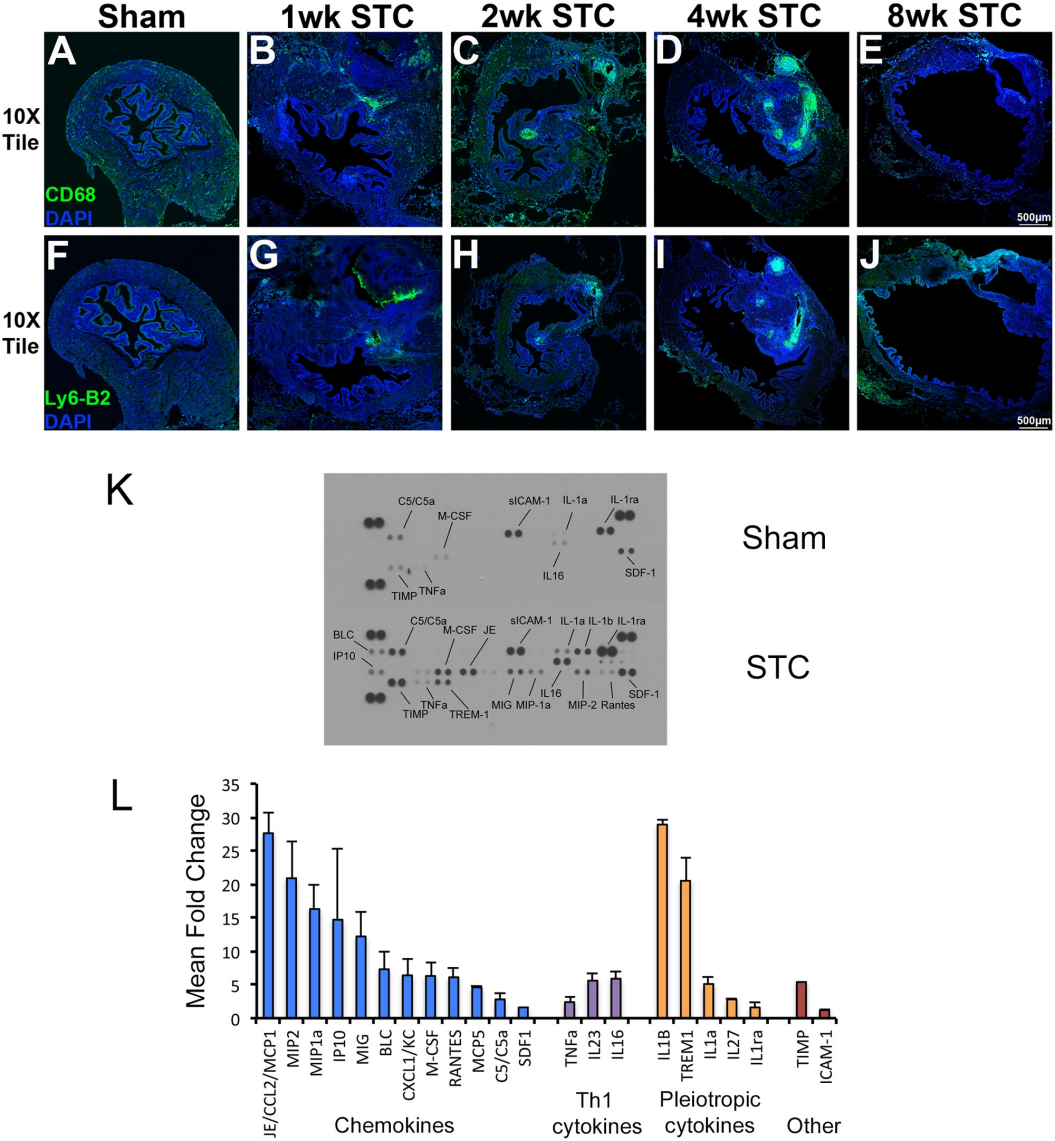
Inflammatory cell invasion into the regenerating portion of the bladder is characterized by a pro-inflammatory cytokine response. Tiled confocal images of bladder sections stained with antibodies to CD68 (A-E) and Ly-6B.2 (F-J). Anti-CD68 marks infiltrating macrophages and anti-Ly-6B.2 marks infiltrating neutrophils. Leftmost panels (A, F) are sham bladders. Other panels are from STC surgery and are labeled as above. (K) Representative images of cytokine arrays interrogated with protein isolated from sham surgery bladders (top) and STC surgery bladders (bottom) one week after surgery. Cytokines of interest are indicated next to each set of duplicate spots. (L) Densitometric quantification of images similar to and including those shown in (K). Only bars that demonstrated statistically significant changes (P < 0.05) are shown. Bars are grouped by color: blue bars represent chemokines, purple bars represent Th1 cytokines, yellow bars represent pleiotropic cytokines and red bars represent TIMP and ICAM-1.

We investigated if genes associated with regeneration, smooth muscle growth and scarring were expressed after STC. Mice were subjected to sham and STC surgery, total RNA was isolated after 1, 2, 4 and 8wk and samples interrogated using qPCR. We first tested RNA for genes encoding extracellular matrix (ECM) proteins or ECM remodeling proteins (Fig 9A). Here we observed that matrix metalloproteinase (MMP) -2 and fibronectin-1 (FN-1) were altered in STC bladders. Both FN-1 and MMP-2 were significantly increased by 2.8-fold relative to shams 1wk after STC. Both genes were increased in STC bladders at 2 and 4wk, but these values were not statistically significant (S2 Table). Thus, at least two genes associated with tissue remodeling during regeneration were increased 1wk after STC. This was not true for the genes encoding ECM components LN5 and TNC which were not significantly altered in STC bladders compared to shams (Fig 8A). We amplified genes expressed in bladder mesenchymal stems to determine if there were changes to this cell population in response to STC. Here we found increased expression of CD34 and Sca-1, with statistical significance found at 4wks for CD34 and at 1 and 4wks for Sca-1. Thus, while trending the same or higher than shams, STC bladders demonstrate changes to a small number of genes associated with regeneration.

Increases in smooth muscle specific gene expression could indicate that there is growth or repair of smooth muscle after STC. We tested our RNA samples for expression of smooth muscle myosin (*SMM*), smooth muscle alpha actin (*ACTA2*), *P2RX1* and serum response factor (*SRF*). We found that at 1wk PS *SMM*, *ACTA2* and *P2RX1* were all decreased relative to the sham, although none of these were statistically significant. *SRF*, on the other hand, remained at the same level as in the sham samples (Fig 8B, S3 Table)). At 2wk *SMM* was decreased relative to shams, *SRF* and *ACTA2* were equivalent to shams and *P2RX1* was increased 3.7-fold, although none were significant. At 4wk *SMM*, *ACTA2* and *P2RX1* were decreased relative to shams and all three were statistically significant. The trend continued at 8wk, where *SMM*, *ACTA2* and *P2RX1* were reduced relative to shams. SRF increased to be 3.8-fold higher than shams, but again this was not statistically significant. Overall, smooth muscle genes appeared to be the same or reduced relative to shams after STC, with few of these data points reaching significance.

To test whether fibrosis or scarring is associated with STC we tested our RNA samples for expression of *Collagen1α1 (Col1α1)*, *Collagen3α1 (Col3α1)*, *Collagen7α1 (Col 7α1)*, *FSP-1*, *Vimentin*, and *Zeb-2*. We observed that all of these genes were significantly increased relative to sham at various times (Fig 8C). Statistically significant increased expression was observed for *Col1α1*at 4wk; *Col3α1* at 1wk and 4wk; *Col7α1* at 1wk, 2wk and 4wk; *FSP-1* at 1wk; *Vimentin* at 1wk and 4wk; and *Zeb-2* at 1wk (Fig 8C, S4 Table). Notably, *Col1α1* and *Col3α1* levels were similar to that of GAPDH, but *Col7α1* levels were much lower (~10^5^-fold lower). In 8wk samples expression of all the genes tested were the same or less and not significantly different than sham operated bladders. Thus, genes associated with fibrosis and scarring were expressed in the first four weeks following STC.

To investigate whether neural or urothelial genes were altered in response to STC we tested our RNA samples for *NeuN* [24], *NF200*, *uroplakin II (UpkII)*, *cytokeratin-5* (*Ck5*) and sonic hedgehog (*SHH*) (Fig 8D, S5 Table). We observed that *NeuN* was decreased after STC, with significance at 1wk. NF200 was increased most prominently at 2wk where it was 13.6-fold higher than sham operated. In fact, apart from *NeuN*, all the genes tested were increased reaching highest levels at 2wk, with all statistically significant except for *UpkII*. These findings support the hypothesis that genes associated with regrowth of nerves and urothelium are expressed after STC.

### The inflammatory response after STC involves both pro- and anti-inflammatory cytokines

Inflammation is thought to play a critical role in regeneration and inflammatory cells are required for salamander limb regeneration [25] and zebrafish heart regeneration [26]. To investigate if inflammatory cells localize to the suture site of the STC bladder we stained sections for CD68 (macrophages) and Ly6B2 (neutrophils). Here we observed a large infiltration of macrophages into the suture site after 1wk (Fig 9A). Macrophages were present in the bladder 2wks and 4wks PS and were about equal to sham operated bladders at 8wks PS. Neutrophils invaded the bladder by 1wk, similar to macrophages. However, the number of Ly6B2 cells was very much reduced by 2wk and there was nominal staining with this antibody in 4wk and 8wk samples.

Influx of macrophages and upregulation of anti-inflammatory cytokines has been shown to play an important role in salamander limb regeneration [25]. This requirement involved the presence of a range of cytokines. We therefore tested regenerating bladders for the presence of cytokines 1wk using protein arrays (Fig 9B). Here we found that a number of cytokines were increased in the bladder after STC. Cytokines involved with inflammation and endothelial activation were increased in the bladder: C5a, TREM-1 and sICAM-1. Chemokines BLC, IP-10, MIP-2 and RANTES were increased but others, CCL-1, CCL-11, I-Tac, KC, MIG and TARC were unchanged (Fig 9C). Th1 cytokines interferon gamma, IL-2, and TNF-b were not altered by STC but TNF-α and IL-16 increased. None of the anti-inflammatory cytokines (Th2) we tested, IL-4, IL-5, IL-6, IL-9, IL-10 or IL-13 were increased 1wk after STC (Fig 9B and 9C). These findings indicate that the inflammatory response to STC is robust, but does not involve an increase in anti-inflammatory cytokines.

## Discussion

An important question facing regenerative medicine is how to activate the intrinsic ability of tissue to regrow as happens in animals such as newts, salamanders and fish. Combining such a process with tissue engineering has the opportunity to heal or regrow organs such as the bladder. Supporting this idea is the knowledge that mammalian tissues from embryos or neonates can regenerate [27]. This suggests that the pathways involved with true regeneration are lost not in mammals but are altered in the transition from embryo to adult. Since the bladder is easily accessed in the abdomen and its function can be easily assessed after surgery, the bladder is an excellent model for studying and manipulating mammalian regeneration in adult mice.

While it has been apparent that the bladder restores its capacity to store urine after cystectomy, it has not been clear if this involved scarring or fibrosis, processes that affect bladder function, such as voiding reflex. Our findings indicate that some pathways that support healthy regeneration are activated after STC, including new muscle growth and angiogenesis. However, these pathways are accompanied by other events that lead to scarring or fibrosis. Thus, bladder regrowth involves simultaneous activation of regeneration and scarring. This understanding allows us to see the unique and important status for urinary bladder in the field of mammalian regeneration, because while imperfect, regeneration occurs and it could now be perfected by introducing interventions such as drugs or gene therapy.

What has not been recognized up to this point in time is that fibrosis and scarring are occurring in the bladder after STC. One reason that scarring mechanisms may be activated after STC is that the smaller bladder creates a low volume, high pressure system. High pressures are also observed after partial bladder outlet obstruction (pBOO) and this leads to non-compliant, fibrotic bladders with increased collagen deposition within the detrusor muscle [28]. When 50–70% of the bladder is removed leaving the ureters intact, there is greater resistance for urine to drain from the kidney into the bladder, as well as increased pressure across the bladder wall. This promotes the formation of collagen fibrils within the detrusor muscle to increase the tensile strength of the wall. In fact, we observed altered maximum voiding volumes and frequency in STC mice, as well as increased collagen by Masson’s trichrome relative to sham (laparotomy) controls. It would therefore be interesting to test if decreasing bladder pressures in the STC bladder would alleviate some of the scarring after STC and create a permissive condition for regeneration.

There have been clues in the literature suggesting that bladder regeneration is associated with scarring or fibrosis. When Burmeister et al. (2010) tested muscle strips from regenerating adult female rat bladders after STC, the authors found that while maximal contractile response (MCR) to and electrical field and pharmacological stimulus increased over time, MCR from STC bladder strips remained lower at all time points compared with age-matched, non-surgical control bladders [19]. Such a finding would be consistent with tissue perturbations in the smooth muscle caused by increased collagen deposition. A later study by Burmeister et al. (2013) showed age differences in the ability of the female rat bladder to regenerate [14]. In this study, detrusor strips from both young (12wk) and old (12mo) bladders contracted less than strips from age-matched, non-surgical controls. Bladders from older rats regenerated less than those from younger rats, were not able to hold as much urine and exhibited quantifiable collagen accumulation. The differences in our analysis may be attributable to differences between rats and mice, but the findings from these studies suggest that scarring or fibrosis is present in the rat model as well.

Another major difference we observed in mice subjected to STC was in the cytokines produced in the bladder. In salamanders, the regenerating limb requires the infiltration of macrophages and release of anti-inflammatory cytokines [25]. Increases in anti-inflammatory Th2 cytokines were observed in the blastema 1-7d after limb amputation. Modulation of the immune response was also observed to alter heart regeneration in zebrafish and the fresh water teleost, medaka. In fish hearts, as in salamander limb, macrophage infiltration was an important component of regeneration. However, if this was inhibited, neutrophils were not rapidly cleared, cardiomyocyte proliferation was reduced and scar tissue persisted. When Toll-like receptors were stimulated, these effects were reversed and regeneration was improved suggesting that stimulating the innate immune system is important for regeneration [26]. We observed that 1wk after STC macrophages and neutrophils invade the bladder and a number of chemokines, Th1 cytokines and pleiotropic cytokines increased in the bladder. Of these, a number of the pro-inflammatory cytokines including TNFα, IL-1α and IL-1β were increased but none of the anti-inflammatory Th2 cytokines we interrogated increased. Increased pro-inflammatory cytokines early after injury followed by increased anti-inflammatory cytokines at later times is typical of wound healing with scarring and not regeneration [29].

In summary, we utilized the STC model in adult female mice to test several parameters of bladder regrowth. We found that the bladder does regrow to restore function as a reservoir for urine. This is accompanied by cell proliferation, cell movement, immune cell infiltration, cytokine expression and fibrosis. Gene expression was characterized by increased expression consistent with regeneration and fibrosis and decreased or constant expression of smooth muscle genes. Thus, we conclude that bladder regeneration is an example of compensatory regrowth not true regeneration. The next step will be to find the key to unlocking true regeneration in the urinary bladder.

## Supporting information

S1 Table Summary of the primary antibodies used for this study with vendor, catalog number, lot number (when available) and dilution factor indicated. (DOCX)

S2 Table Table indicating data for ΔCT values, standard error of the mean and P-values for stem cell and extracellular matrix genes tested in this study. Statistical significance is indicated by an * when P < 0.05.
(DOCX)

S3 Table Table indicating data for ΔCT values, standard error of the mean and P-values for smooth muscle genes tested in this study. Statistical significance is indicated by an * when P < 0.05.
(DOCX)

S4 Table Table indicating data for ΔCT values, standard error of the mean and P-values for fibrosis associated genes tested in this study. Statistical significance is indicated by an * when P < 0.05.
(DOCX)

S5 Table Table indicating data for ΔCT values, standard error of the mean and P-values for neuronal and urothelial genes tested in this study. Statistical significance is indicated by an * when P < 0.05.
(DOCX)

S1 Fig Cystometry of sham operated and STC bladders. (A, B) Graphs showing representative data from cytometry analysis. The y-axis is voiding pressure in cm H_2_O. The x-axis is elapsed time in minutes. (A) Representative sham operated bladder and (B) representative STC bladder. (C) Summary of results of voiding pressure in cm H_2_O. Here sham and STC operated bladders are from mice aged 1-4wk post-surgery.
(TIF)

S2 Fig Staining for keratin-14 in bladders 1wk following sham, linear incision and STC surgery. (A-C) Cryosections were stained with an antibody to keratin-14 to identify activated urothelial cells (green). Type of surgery is indicated over each panel. White arrowheads point to representative positive cells. Sections were counterstained in blue (DAPI). Magnification is indicated at the bottom of each panel.
(TIF)

S3 Fig Staining for phospho histone H3 in bladders following sham and STC surgery. Top row of panels: tiled confocal images of sham operated and STC operated bladders 1wk, 2wk, 4wk and 8wk after surgery. These sections were stained with an antibody to phospho histone H3 to identify cells undergoing cell division at that moment (red). Bottom row are higher magnifications of the areas inside the white frames in the panel directly above. Here cells reacting with anti-phospho histone H3 are more apparent (red). Sections were counterstained in blue (DAPI). Magnification is indicated in the two leftmost panels for each row.
(TIF)

S4 Fig Staining for Ki-67 in bladders 1wk following sham and STC surgery. (A-C) Cryosections were stained with an antibody to Ki-67 to identify cells undergoing cell division at that moment (red). Here there are more cells reacting (red) with anti-Ki-67 after STC than in sham (B). This increase was observed both in urothelium (B) and in the detrusor layer (C). White arrowheads indicate the location of some of these nuclei. Sections were counterstained in blue (DAPI). Magnification is indicated at the bottom of each panel.
(TIF)
