# Supplementary material for: Compensatory regrowth of the mouse bladder after partial cystectomy
Source: PLoS One. 2018 Nov 26;13(11):e0206436. doi: 10.1371/journal.pone.0206436 (PMC6261052; doi:10.1371/journal.pone.0206436)
Supplement: S1 Table — (DOCX) [file pone.0206436.s001.docx]

| Primary Antibody Name | Vendor | Catalog Number | Lot Number | Dilution |
| --- | --- | --- | --- | --- |
| Cytokeratin-5 | Abcam | Ab52635 | GR157618-5 | 1:100 |
| Smooth Muscle Myosin | Alfa Aesar | J64817 | 5620715 | 1:100 |
| Smooth Muscle Actin | Sigma | A5228-200uL | 074M4814V | 1:100 |
| Phospho-Histone H3 | Millipore | 06-570 | DAM1713192 | 1:100 |
| Sca-1 | BD Biosciences | 557403 | 5163593 | 1:100 |
| CD34 | eBioscience | 14-0341-82 | E03672-1632 | 1:100 |
| Ly6-B2 | Bio-Rad | MCA771GA | Clone 7/4 | 1:100 |
| UPK2 (n-18) | Santa Cruz | Sc-15178 | E2909 | 1:100 |
| F4/80 (macrophage) | Biolegend | 123101 | B197364 | 1:100 |
| CD68 (macrophage) | Biolegend | 137001 | B208956 | 1:100 |
| Cytokeratin-14 | Biolegend | 906001 |  | 1:250 |
| Ki67 | Abcam | ab15580 |  | 1:150 |
