# Supplementary material for: Compensatory regrowth of the mouse bladder after partial cystectomy
Source: PLoS One. 2018 Nov 26;13(11):e0206436. doi: 10.1371/journal.pone.0206436 (PMC6261052; doi:10.1371/journal.pone.0206436)
Supplement: S2 Table — Statistical significance is indicated by an * when P < 0.05. (DOCX) [file pone.0206436.s002.docx]

| Gene | ΔCT Sham | ΔCT Sham | SEM Sham | SEM STC | P-value |
| --- | --- | --- | --- | --- | --- |
| *CD34 1wk* | 3.80 | 2.87 | 0.30 | 0.22 | 0.065 |
| *CD34 2wk* | 2.99 | 3.97 | 0.34 | 1.32 | 0.51 |
| *CD34 4wk* | 3.94 | 2.73 | 0.23 | 0.31 | 0.034* |
| *CD34 8wk* | 4.01 | 4.23 | 0.17 | 0.52 | 0.71 |
| *SCA-1 1wk* | 3.80 | 2.73 | 0.08 | 0.27 | 0.017* |
| *SCA-1 2wk* | 5.54 | 4.71 | 0.12 | 1.10 | 0.49 |
| *SCA-1 4wk* | 4.39 | 2.93 | 0.22 | 0.20 | 0.0071* |
| *SCA-1 8wk* | 4.95 | 4.67 | 0.34 | 0.43 | 0.63 |
| *FN-1 1wk* | 0.85 | -0.64 | 0.12 | 0.04 | 0.00028* |
| *FN-1 2wk* | 2.16 | 0.56 | 0.84 | 0.78 | 0.23 |
| *FN-1 4wk* | -0.26 | -1.66 | 1.09 | 0.59 | 0.32 |
| *FN-1 8wk* | 0.30 | 0.52 | 0.27 | 0.47 | 0.71 |
| *MMP-2 1wk* | 2.79 | 1.30 | 0.18 | 0.30 | 0.013* |
| *MMP-2 2wk* | 4.96 | 3.63 | 0.29 | 0.73 | 0.17 |
| *MMP-2 4wk* | 3.34 | 2.34 | 0.01 | 0.64 | 0.19 |
| *MMP-2 8wk* | 4.37 | 4.36 | 0.18 | 0.56 | 0.99 |
| *LN-5 1wk* | 4.98 | 5.02 | 0.07 | 0.42 | 0.92 |
| *LN-5 2wk* | 8.36 | 7.03 | 0.71 | 1.06 | 0.35 |
| *LN-5 4wk* | 5.73 | 5.38 | 0.48 | 0.12 | 0.52 |
| *LN-5 8wk* | 5.78 | 5.58 | 0.45 | 2.24 | 0.34 |
| *TNC 1wk* | 2.88 | 2.47 | 0.04 | 0.20 | 0.11 |
| *TNC 2wk* | 3.88 | 4.03 | 0.39 | 1.08 | 0.90 |
| *TNC 4wk* | 4.02 | 3.97 | 0.43 | 0.64 | 0.95 |
| *TNC 8wk* | 3.29 | 3.68 | 0.15 | 1.48 | 0.54 |
