# Supplementary material for: Compensatory regrowth of the mouse bladder after partial cystectomy
Source: PLoS One. 2018 Nov 26;13(11):e0206436. doi: 10.1371/journal.pone.0206436 (PMC6261052; doi:10.1371/journal.pone.0206436)
Supplement: S3 Table — Statistical significance is indicated by an * when P < 0.05. (DOCX) [file pone.0206436.s003.docx]

| Gene | ΔCt Sham | ΔCt STC | SEM Sham | SEM STC | P value |
| --- | --- | --- | --- | --- | --- |
| *SMM 1wk* | 1.00 | 3.7 | 0.75 | 1.49 | 0.66 |
| *SMM 2wk* | 0.99 | 2.2 | 0.58 | 1.3 | 0.44 |
| *SMM 4wk* | -0.91 | 0.30 | -0.3 | 0.16 | 0.02* |
| *SMM 8wk* | -1.15 | 0,07 | -0.23 | 0.89 | 0.26 |
| *SRF 1wk* | 3.84 | 3.99 | 0.22 | 0.17 | 0.61 |
| *SRF 2wk* | 5.48 | 5.83 | 0.23 | 1.11 | 0.77 |
| *SRF 4wk* | 4.72 | 5.12 | 0.27 | 0.20 | 0.29 |
| *SRF 8wk* | 5.25 | 3.33 | 0.08 | 0.24 | 0.37 |
| *ACTA2 1wk* | -4.83 | -1.98 | -.1.13 | -0.40 | 0.08 |
| *ACTA2 2wk* | 0.21 | -0.21 | 0.62 | -1.32 | 0.79 |
| *ACTA2 4wk* | -1.61 | -0.47 | -0.02 | -0.18 | 0.0034* |
| *ACTA2 8wk* | -1.95 | -0.74 | -0.13 | -0.92 | 0.26 |
| *P2RX1 1wk* | 2.63 | 4.55 | 0.15 | 0.70 | 0.055 |
| *P2RX1 2wk* | 7.53 | 5.65 | 1.33 | 0.40 | 0.24 |
| *P2RX1 4wk* | 1.63 | 3.4 | 0.25 | 0.11 | 0.0029* |
| *P2RX1 8wk* | 1.59 | 2.7 | 0.49 | 0.73 | 0.27 |
