# Supplementary material for: Compensatory regrowth of the mouse bladder after partial cystectomy
Source: PLoS One. 2018 Nov 26;13(11):e0206436. doi: 10.1371/journal.pone.0206436 (PMC6261052; doi:10.1371/journal.pone.0206436)
Supplement: S4 Table — Statistical significance is indicated by an * when P < 0.05. (DOCX) [file pone.0206436.s004.docx]

| Gene | ΔCT Sham | ΔCT STC | SEM Sham | SEM STC | P-value |
| --- | --- | --- | --- | --- | --- |
| *Col1α1 1wk* | -2.79 | -5.19 | 0.56 | 0.73 | 0.059 |
| *Col1α1 2wk* | 0.95 | -1.73 | 0.29 | 1.12 | 0.082 |
| *Col1α1 4wk* | 0.21 | -1.27 | 0.16 | 0.10 | 0.0013* |
| *Col1α1 8wk* | -1.16 | -0.82 | 1.14 | 0.56 | 0.81 |
| *Col3α1 1wk* | -0.17 | -2.41 | 0.33 | 0.21 | 0.0045* |
| *Col3α1 2wk* | 1.98 | -0.23 | 0.29 | 0.96 | 0.091 |
| *Col3α1 4wk* | 1.30 | -0.39 | 0.30 | 0.20 | 0.0097* |
| *Col3α1 8wk* | 1.13 | 0.58 | 0.04 | 0.52 | 0.35 |
| *Col7α1 1wk* | 11.21 | 8.69 | 0.22 | 0.41 | 0.0055* |
| *Col7α1 2wk* | 14.80 | 11.35 | 0.10 | 0.65 | 0.0062* |
| *Col7α1 4wk* | 12.33 | 10.21 | 0.67 | 0.35 | 0.048* |
| *Col7α1 8wk* | 12.22 | 11.19 | 0.23 | 0.56 | 0.17 |
| *FSP-1 1wk* | 6.81 | 5.03 | 0.21 | 0.15 | 0.0022* |
| *FSP-1 2wk* | 8.46 | 6.49 | 0.06 | 0.90 | 0.094 |
| *FSP-1 4wk* | 6.49 | 5.68 | 0.36 | 0.13 | 0.10 |
| *FSP-1 8wk* | 6.17 | 6.28 | 0.83 | 0.21 | 0.90 |
| *Vimentin 1wk* | 2.40 | 0.51 | 0.35 | 0.057 | 0.0063* |
| *Vimentin 2wk* | 3.75 | 2.26 | 0.27 | 0.92 | 0.19 |
| *Vimentin 4wk* | 3.70 | 1.82 | 0.15 | 0.38 | 0.010* |
| *Vimentin 8wk* | 2.97 | 1.75 | 0.41 | 0.012 | 0.57 |
| *Zeb2 1wk* | 3.55 | 2.86 | 3.55 | 2.86 | 0.032* |
| *Zeb2 2wk* | 4.41 | 3.25 | 0.28 | 0.40 | 0.074 |
| *Zeb2 4wk* | 3.96 | 3.59 | 0.20 | 0.35 | 0.42 |
| *Zeb2 8wk* | 3.62 | 3.72 | 0.59 | 0.09 | 0.87 |
