# Supplementary material for: Compensatory regrowth of the mouse bladder after partial cystectomy
Source: PLoS One. 2018 Nov 26;13(11):e0206436. doi: 10.1371/journal.pone.0206436 (PMC6261052; doi:10.1371/journal.pone.0206436)
Supplement: S5 Table — Statistical significance is indicated by an * when P < 0.05. (DOCX) [file pone.0206436.s005.docx]

| Gene | ΔCT Sham | ΔCT STC | SEM Sham | SEM STC | P-value |
| --- | --- | --- | --- | --- | --- |
| *Neun 1wk* | 4.95 | 6.35 | 0.13 | 0.40 | 0.028* |
| *Neun 2wk* | 5.43 | 7.55 | 0.30 | 1.08 | 0.13 |
| *Neun 4wk* | 4.60 | 5.98 | 0.11 | 0.60 | 0.085 |
| *Neun 8wk* | 4.57 | 5.07 | 0.28 | 0.73 | 0.59 |
| *NF200 1wk* | 10.65 | 10.44 | 0.40 | 0.28 | 0.69 |
| *NF200 2wk* | 13.55 | 10.44 | 0.95 | 0.41 | 0.039* |
| *NF200 4wk* | 12.02 | 10.42 | 0.80 | 0.53 | 0.17 |
| *NF200 8wk* | 8.89 | 10.39 | 0.95 | 0.41 | 0.21 |
| *UPKII 1wk* | -0.64 | 0.82 | 0.03 | 0.10 | 0.00018* |
| *UPKII 2wk* | 3.69 | 0.63 | 1.11 | 0.39 | 0.059 |
| *UPKII 4wk* | 1.10 | 0.76 | 0.03 | 0.10 | 0.64 |
| *UPKII 8wk* | 1.50 | 0.74 | 0.20 | 0.45 | 0.15 |
| *KRT5 1wk* | 4.67 | 4.30 | 0.10 | 0.22 | 0.195 |
| *KRT5 2wk* | 7.91 | 4.94 | 0.67 | 0.50 | 0.024* |
| *KRT5 4wk* | 4.66 | 4.20 | 0.79 | 0.57 | 0.66 |
| *KRT5 8wk* | 5.15 | 4.69 | 0.75 | 0.28 | 0.69 |
| *SHH 1wk* | 5.72 | 6.01 | 0.09 | 0.08 | 0.069 |
| *SHH 2wk* | 8.48 | 6.00 | 0.60 | 0.34 | 0.023* |
| *SHH 4wk* | 5.83 | 5.38 | 0.63 | 0.33 | 0.56 |
| *SHH 8wk* | 4.83 | 3.67 | 1.01 | 0.41 | 0.48 |
