# Supplementary figures and images for: Compensatory regrowth of the mouse bladder after partial cystectomy
Source: PLoS One. 2018 Nov 26;13(11):e0206436. doi: 10.1371/journal.pone.0206436 (PMC6261052; doi:10.1371/journal.pone.0206436)

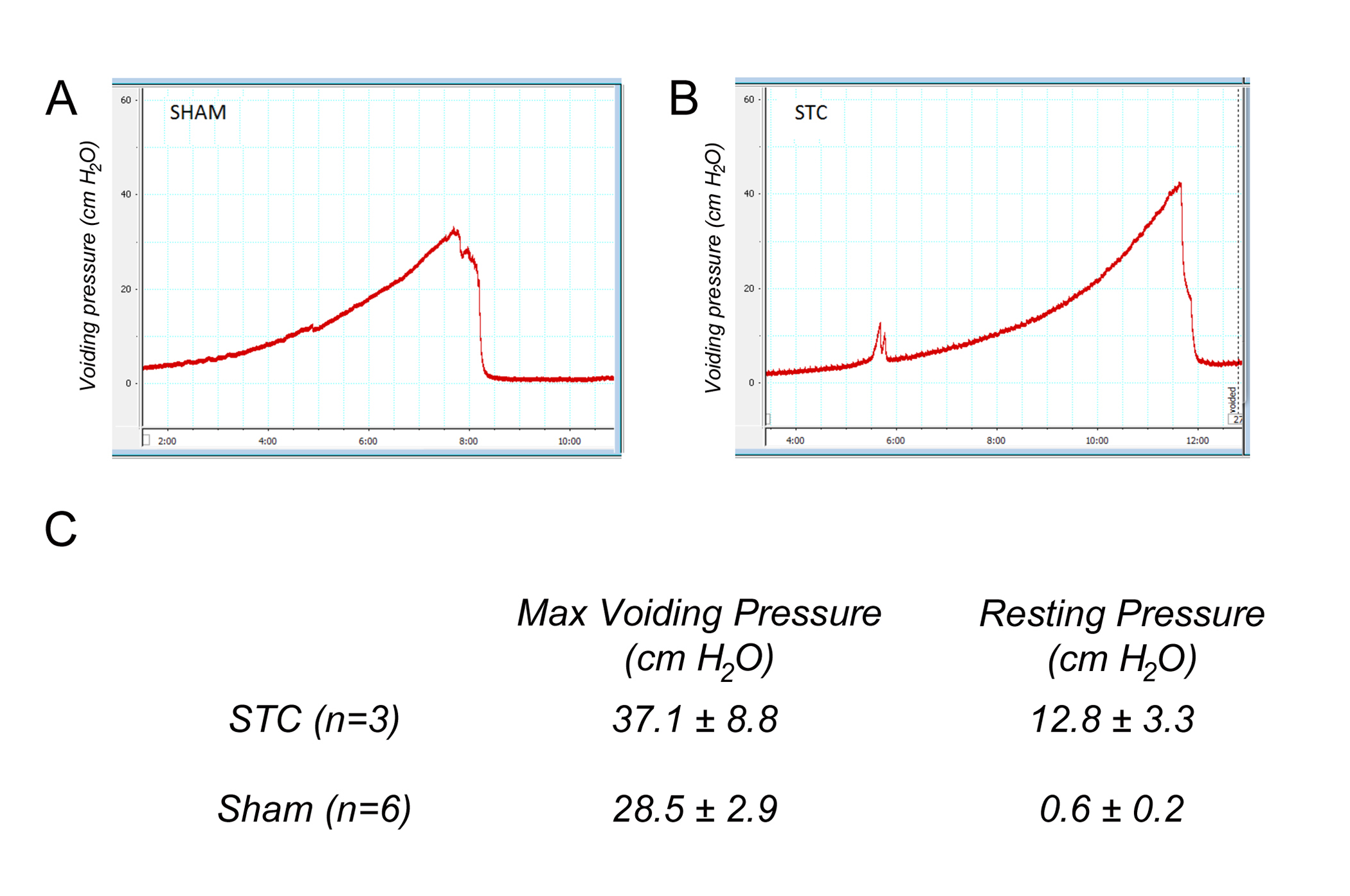

Supplement: S1 Fig — (A, B) Graphs showing representative data from cytometry analysis. The y-axis is voiding pressure in cm H2O. The x-axis is elapsed time in minutes. (A) Representative sham operated bladder and (B) representative STC bladder. (C) Summary of results of voiding pressure in cm H2O. Here sham and STC operated bladders are from mice aged 1-4wk post-surgery. (TIF) [file pone.0206436.s006.tif]

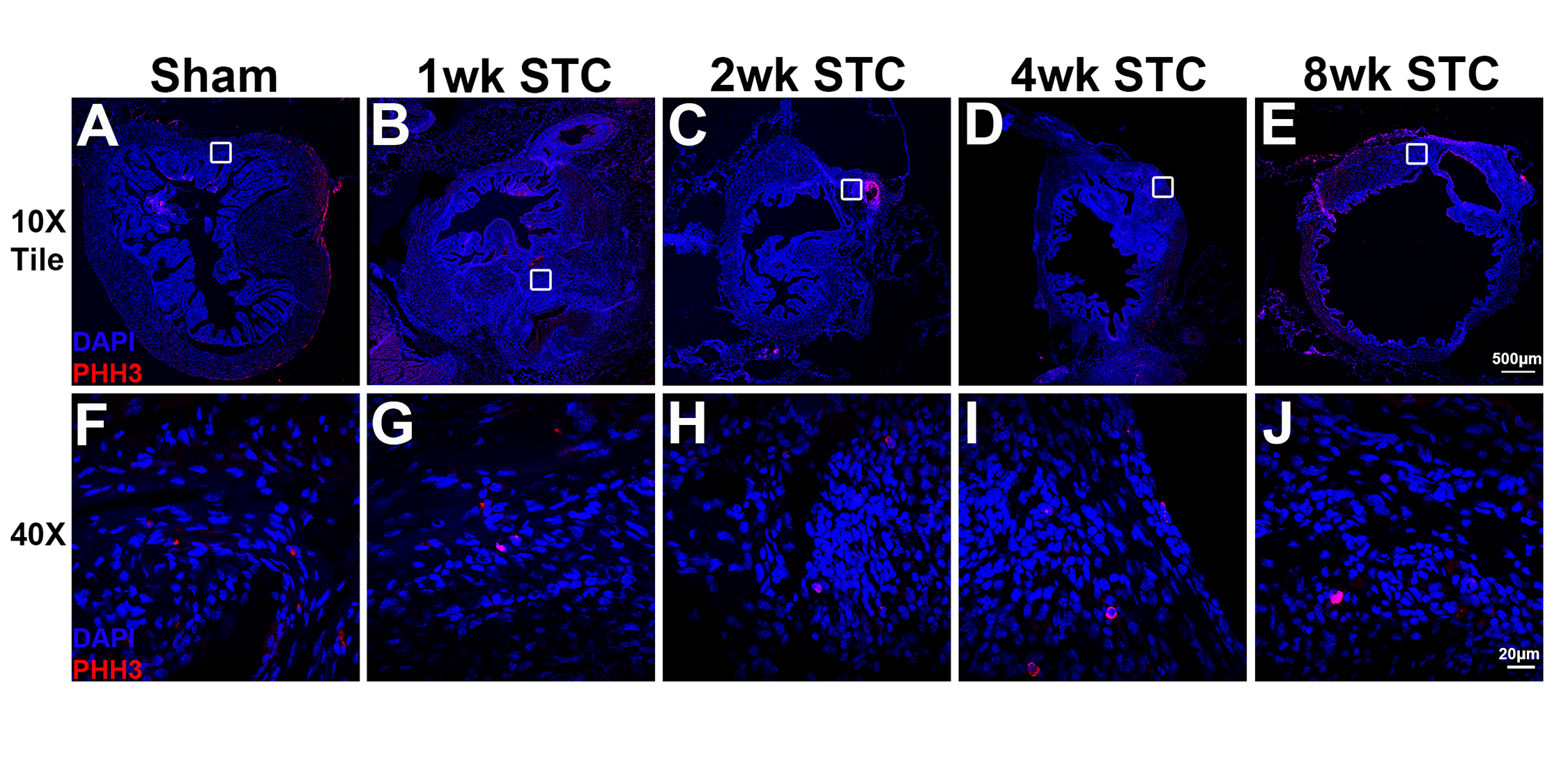

Supplement: S2 Fig — (A-C) Cryosections were stained with an antibody to keratin-14 to identify activated urothelial cells (green). Type of surgery is indicated over each panel. White arrowheads point to representative positive cells. Sections were counterstained in blue (DAPI). Magnification is indicated at the bottom of each panel. (TIF) [file pone.0206436.s007.tif]

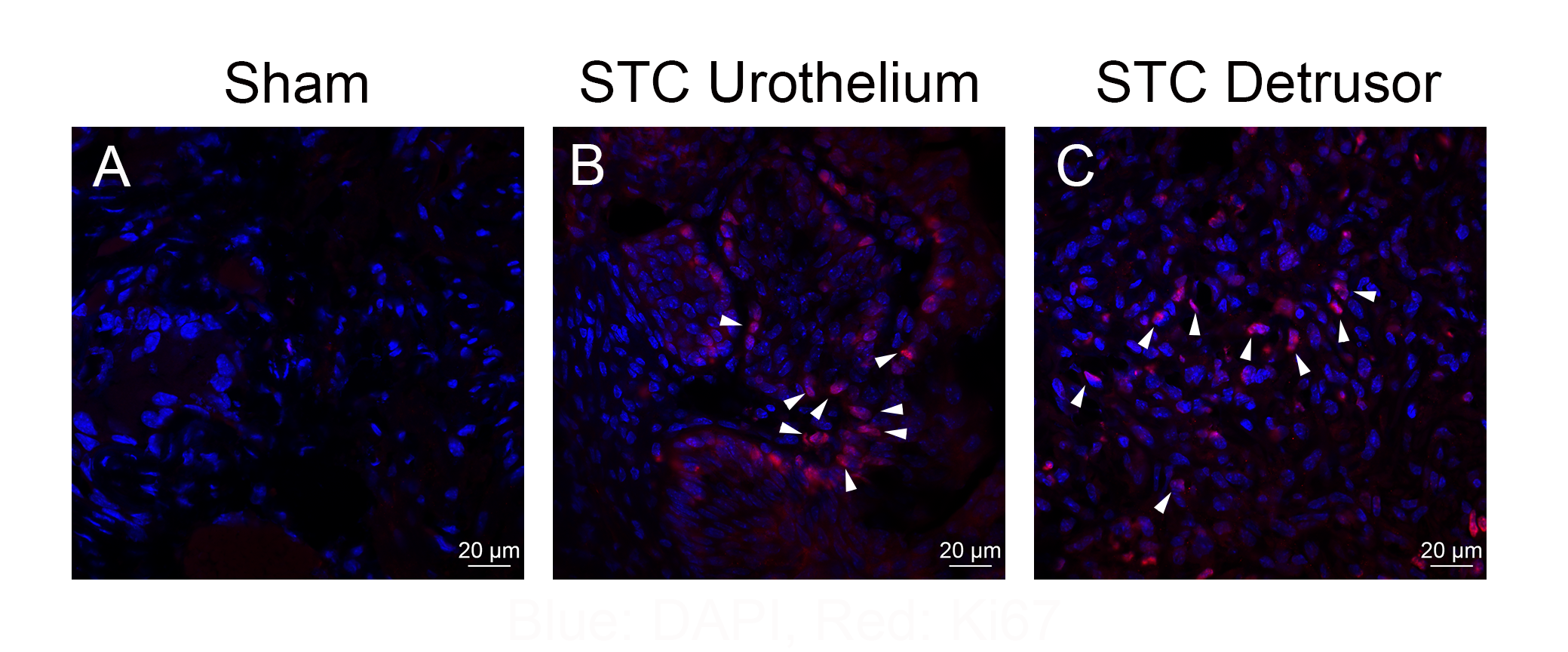

Supplement: S3 Fig — Top row of panels: tiled confocal images of sham operated and STC operated bladders 1wk, 2wk, 4wk and 8wk after surgery. These sections were stained with an antibody to phospho histone H3 to identify cells undergoing cell division at that moment (red). Bottom row are higher magnifications of the areas inside the white frames in the panel directly above. Here cells reacting with anti-phospho histone H3 are more apparent (red). Sections were counterstained in blue (DAPI). Magnification is indicated in the two leftmost panels for each row. (TIF) [file pone.0206436.s008.tif]

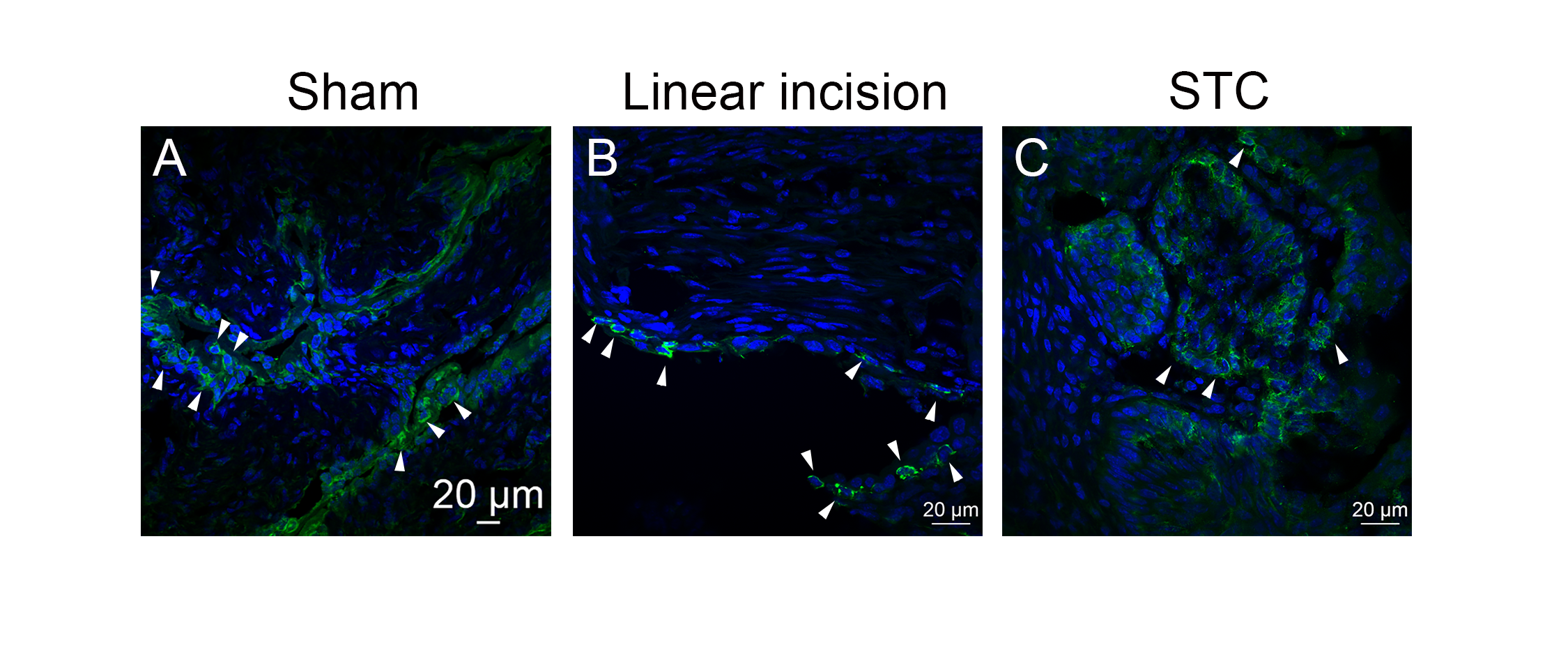

Supplement: S4 Fig — (A-C) Cryosections were stained with an antibody to Ki-67 to identify cells undergoing cell division at that moment (red). Here there are more cells reacting (red) with anti-Ki-67 after STC than in sham (B). This increase was observed both in urothelium (B) and in the detrusor layer (C). White arrowheads indicate the location of some of these nuclei. Sections were counterstained in blue (DAPI). Magnification is indicated at the bottom of each panel. (TIF) [file pone.0206436.s009.tif]
